# A Novel Designed Sandwich ELISA for the Detection of *Echinococcus granulosus* Antigen in Camels for Diagnosis of Cystic Echinococcosis

**DOI:** 10.3390/tropicalmed8080400

**Published:** 2023-08-06

**Authors:** Nagwa I. Toaleb, Dina Aboelsoued, Kadria N. Abdel Megeed, Sahar Hussein Abdalla Hekal

**Affiliations:** 1 Department of Parasitology and Animal Diseases, Veterinary Research Institute, National Research Centre, El Buhouth Street, Dokki, Cairo 12622, Egypt; ni.toaleb@nrc.sci.eg (N.I.T.); kn.nasr@nrc.sci.eg (K.N.A.M.); 2 Department of Natural Resources, Faculty of African Postgraduate Studies, Cairo University, Giza 12613, Egypt; saharhekal@cu.edu.eg

**Keywords:** *Echinococcus granulosus*, PCR, NAD 1 gene, protein A affinity chromatography, polyclonal antibodies, capture ELISA, sandwich ELISA, camel

## Abstract

*Echinococcus* spp. are important cosmopolitan zoonotic parasitic tapeworms that cause a disease called hydatidosis or cystic echinococcosis (CE), which has remarkable economic losses. The objective of our study was to develop a specific IgG polyclonal antigen-based ELISA (Sandwich ELISA; capture ELISA) method for the detection of circulating *Echinococcus granulosus* (*E. granulosus*) antigens in camels infected with hydatid cysts before slaughtering and its application in serodiagnosis of CE in animals to assess the positive rate of hydatidosis in camels slaughtered in Giza governorate abattoirs in Egypt. In this study, molecular identification of *Echinococcus* sp. isolate was performed based on the NADH dehydrogenase subunit 1 (NAD1) gene, revealing the isolate (GenBank: OQ443068.1), which is identical to the G6 *E. granulosus sensu lato* genotype. The positive rate of hydatid cysts was determined in slaughtered camels’ organs (*n* = 587). The results revealed that hydatid cysts were found in 46.5% (273/587) of the examined camels. Pulmonary echinococcosis was significantly more prevalent in the slaughtered camels (60%, 164/273) than hepatic echinococcosis (39.9%, 109/273), (*p* = 0.001, Chi Square = 11.081). Cyst fertility rates were higher in hepatic (90.8%, 99/109) than in pulmonary cysts (83.5%, 137/164) and the most viable protoscoleces were recorded from fertile the hepatic cysts (67.85 ± 12.78). In this study, hydatid cyst germinal layer antigen (GlAg) was isolated and used for the immunization of rabbits to raise IgG polyclonal antibodies (anti-*Echinococcus* GlAb IgG). These IgG polyclonal antibodies were purified by affinity chromatography using a protein A column, then labeled with horseradish peroxidase. Electrophoretic analysis of IgG polyclonal antibodies and crude GlAg was performed in 10% polyacrylamide gels. The SDS-PAGE revealed four bands at molecular weights of 77 kDa, 65 kDa, 55 kDa, and 25 kDa. The Sandwich ELISA was performed to evaluate the sensitivity and specificity and cross-reactivity of the prepared IgG polyclonal antibodies. The circulating hydatid antigen was found in 270 out of the 273 samples with hydatidosis, with a sensitivity of 98.9% (270/273), a specificity of 94.9% (296/312) and a diagnostic efficacy of 96.8%. Regarding the cross reactivity, anti-*Echinococcus* GlAb IgG showed a low cross-reactivity with *Fasciola gigantica* infected camel sera (3/8), and Myiasis (*Cephalopina titillator* larvae; 3/20). No cross-reactivity was recorded with uninfected camel sera (negative sera for *E. granulosus*), and no cross-reactivity was found with antigens of *Eimeria* spp., *Toxoplasma gondii*, *Cryptosporidium* sp., and *Hyalomma dromedarii* (ticks’ infestation). Then, Sandwich ELISA was conducted again to detect *E. granulosus* antigen in all the collected camel sera, which resulted in a 48.7% (286/587) positive rate of CE compared to 46.5% (273/587) using a postmortem inspection (PM diagnosis) (*p* = 0.5, Chi Square = 0.302). In conclusion, the Sandwich ELISA technique introduced in this study appears to be a sufficiently sensitive diagnostic assay for the detection of camels’ echinococcosis using anti-*Echinococcus* GlAb IgG. In addition, it might offer a significant medical and veterinary importance in helping the early detection of hydatidosis, as well as its early treatment.

## 1. Introduction

*Echinococcus granulosus* (*E. granulosus*) is a widely distributed zoonotic cestode, which harbors in the intestines of canids (definitive host) [1]. The infection of humans and many species of domestic and wild animals (intermediate hosts) happens in its larval stage (hydatid cyst), causing a silent disease called cystic echinococcosis (CE) [2,3]. The intermediate host infection, produced after ingestion of contaminated food or water with parasite eggs, is where metacestodes develop as fluid-filled cysts in the lungs, liver, and other organs [3]. CE has a worldwide distribution, variable geographic incidence [4], clinical manifestation [5], and is of an economic and public health importance [6,7]. It is asymptomatic in animals and its diagnosis is performed at necropsy [8]. The annual losses due to hydatidosis have been estimated at about USD 3 billion annually [9], in terms of quantity and quality of milk, meat, wool, decreased fertility, retarded growth, and carcass condemnation [10]. The gross detected pathological lesions in edible organs were in hearts (8%), kidneys (21.3%), lungs (44.6%), livers (17.9%), and spleens (8.2%), and these infected organs represent a significant economic loss to traders and the livestock industry [11]. CE is included as a part of the World Health Organization (WHO) strategic plan [12]. The positive rates of camel hydatidosis were 5.94% in Tunisia [13], 2.7% in Libya [14], 59% and 29.7% in Sudan [15,16], 32.8% in Saudi Arabia [17], and 29.1% in Kenya [18]. In Egypt, CE is endemic in humans and animals [19] with variable positive rates of hydatid cyst, in cattle, sheep, goats, and camels [20]. The positive rate of hydatid cysts among slaughtered camels was 3.7% in Sharkia [21], 5.6% in Giza [22], 9% in Assuit [23], 10% in Aswan [24], 21.7% in Cairo [25], and 39.5% in Beni-Suef [22]. Its control is achieved using long-term strategies of prevention and control, mainly targeted at deworming dogs, meat inspection, health education, and surveillance in both livestock and human populations [26].

Immunodiagnosis remains an important tool for the diagnosis of CE in which different assays have been developed and used for the detection of specific antibodies in serum samples with variable results [27,28,29]. However, detection of serum antibodies has a major drawback as the demonstration of specific antibodies against the hydatid antigen cannot differentiate between recent and past infections [30,31] as the circulating antibodies persist even after clinical or parasitological treatments [32]. In addition, unsatisfactory performances shown by many commercially available immunoassays might be due to the poor quality of the prepared antigens [33,34] as a successful immunodiagnostic test depends on the use of highly specific and sensitive antigens, and the detection of the appropriate antibody class or subclass [28,29]. Enzyme-linked immunosorbent assay (ELISA) is used for the diagnosis of hydatidosis in human and animals [29,35].

The detection of circulating antigens in serum and other bodily fluids has been described in many parasitic infections, including CE, as they are present during the active infection, and their levels continue to decrease after the surgical removal of the hydatid cyst or successful chemotherapy. Therefore, the detection of hydatid circulating antigens might be more useful than the detection of the antibodies in the diagnosis of active or recent CE [36,37], which could provide a definite parasitic diagnosis [38]. There are several available immunoassays for hydatid circulating antigen detection in serum, including ELISAs [37,39], countercurrent immunoelectrophoresis, and a co-agglutination (Co-A) test [40].

The present study aimed to assess the usefulness of a standardized and evaluated Sandwich ELISA in the diagnosis of CE. The development of a specific and simple antigen-based ELISA method for the diagnosis of CE could help in the early detection of *Echinococcus* sp. in camels and give an insight into the detection of CE in other animals and humans.

## 2. Materials and Methods

### 2.1. Ethical Approval

All experimental procedures were performed according to the institutional guidelines of the National Research Centre’s Animal Research Committee under protocol number: 2180212023.

### 2.2. Study Area

A cross-sectional study was conducted between October 2022 and July 2023 on slaughtered camels at the main abattoirs of Giza (Nahia and El-moneb; 29°58′27.00″ N, 31°08′2.21″ E) Giza, Egypt.

### 2.3. Animals

Five hundred and eighty-seven camels (530 old and 57 young) were examined before and after slaughtering at the main Giza abattoirs and screened for the presence of hydatid cysts and other infectious diseases in the camels’ organs.

### 2.4. Samples

#### 2.4.1. Blood

Five hundred and eighty-seven (587) blood samples were collected from the camels during the veterinary medical examinations in the abattoir by restraining the camels in a laying down position (with the assistance of two camel handlers) and drawing 5 milliliters of whole blood from the jugular vein using a sterile needle [41].

The blood samples were divided after postmortem (PM) inspection and laboratory examination as described in Table 1. The blood samples were allowed to clot for serum separation and then the sera were stored at −20 °C until use.

#### 2.4.2. Hydatid Cysts

Five hundred and eighty-seven camels were examined after slaughter from the main abattoirs at Giza and screened for the presence of hydatid cysts and other infections in the camels’ organs. Lungs, livers, and other organs of every animal were examined visually and by palpation for the detection of hydatid cysts [42]. The infected organs were collected, and the hydatid cysts were separated in phosphate buffered saline (PBS, pH = 7.2).

### 2.5. Assessment of Fertility and Viability of the Hydatid Cysts

Large liver and lung hydatid cysts were collected from 273 naturally infected slaughtered camels. The cysts were washed several times in PBS (pH = 7.2). The outer surfaces of the cysts were sterilized using 70% ethyl alcohol. The hydatid cyst fluid was collected as described by Smyth [43] and the fertility of the cysts was determined by the presence of protoscoleces in the cystic fluid by microscopical (Olympus Microscope, Model CX41, Olympus Corporation, Japan) examination of a wet mount drop. The fluid was centrifuged at 5000 rpm for 5 min and the pellet was observed at 40× magnification for the presence of protoscoleces. Cyst viability was assessed using the eosin exclusion method as described by Daryani et al. [44].

### 2.6. Separation of Germinal Layers

Germinal layers (inner layers) of large cysts containing viable protoscoleces were separated carefully from the outer layers (laminated layer) using forceps according to Hassanain et al. [29]. Then, the separated germinal layers were examined microscopically to confirm the absence of any traces of the outer layer, washed extensively with PBS (pH = 7.2), and stored at −20 °C until use [29].

### 2.7. DNA Extraction

Genomic DNA was extracted from protoscoleces of 10 camel liver cysts using the QIAamp DNA mini kit (QIAGEN, Cat. No. 51304, Hilden, Germany), following the manufacturer’s instructions. The DNA concentrations were assessed by microvolume spectrophotometer (Q9000, Quawell, Beijing, China). The DNA was stored at −20 °C.

### 2.8. Polymerase Chain Reaction (PCR) and Electrophoresis

PCR was performed on the extracted DNA using primers targeting 500 bp fragment of NADH dehydrogenase subunit 1 (NAD1) gene (F:5′-AGATTCGTAAGGGGCCTAATA-3′ and R:5′-ACCACTAACTAATTCACTTTC-3′) according to Aboelhadid et al. [45] using a thermal Cycler (Model T100, BIO-RAD, Singapore). The PCR products were visualized using a Molecular Imager (Gel Doc™ XR+, Bio-Rad, Hercules, California, USA) in 1.5% agarose gel electrophoresis, stained with RedSafe (Intron Biotechnology, Seongnam, Gyeonggi-do, Republic of Korea), and estimated by a 100 bp ladder (QIAGEN, Cat. No. 239035, USA).

### 2.9. Sequencing and Phylogenetic Analysis

To purify the PCR products positive for *Echinococcus* sp., a gel extraction kit (GeneDirex, Cat. No. NA006-0100, Taoyuan City, Taiwan) was used according to the manufacturer’s protocol. The purified PCR products were sequenced using an automated sequencer (ABI 3130, Applied Biosystems, Waltham, Massachusetts, USA) using a Big Dye Terminator v3.1 Cycle Sequencing Kit (Applied Biosystems, USA). The obtained sequences were corrected by ChromasPro 1.7 software (Technelysium Pty Ltd., South Brisbane, QLD, Australia) then compared using BLASTn (https://blast.ncbi.nlm.nih.gov/Blast.cgi (accessed on 16 February 2023) with the sequences available in GenBank, and submitted to GenBank. Then, multiple sequence alignments were performed using CLUSTAL W v1.83 in the MegAlign module of the Lasergene software package (DNASTAR, Madison, WI, USA). A phylogenetic analysis was performed using MEGA6 software [46].

### 2.10. Preparation of Hydatid Cyst Germinal Layer Antigen (GlAg)

The GlAg of *E. granulosus* (confirmed by PCR as described earlier) was prepared by homogenizing the germinal layer in 0.15 M PBS (pH = 7.2) using glass homogenizer at 4 °C. The homogenate was sonicated for 12 cycles of 30 s each 100 m Amp by 150 ultra-sonication (Sanyo Gallen Kamp PLC, Heathfield, East Sussex, UK), and centrifuged at 16,000 rpm for 30 min at 4 °C. The protein content of the supernatant collected was estimated using the method described by Lowry et al. [47].

### 2.11. Rabbit IgG Polyclonal Antibodies (Anti-Echinococcus GlAb)

Two healthy male New Zealand rabbits about 2 months of age and weighting about 1.5–2 Kg (examined daily for 2 weeks to ensure they are parasite free) were used. The IgG polyclonal antibodies were raised against specific *E. granulosus* GlAg, according to Goubodia and Fagbemi [48] with some modifications. The blood samples were collected from the two rabbits before injection and after immunization according to Engvall and Perlmann [49].

### 2.12. Purification of Rabbit IgG Polyclonal Antibodies Using Protein A Affinity Chromatography

Two purification procedures were undertaken: Firstly, ammonium sulfate precipitation was used, in which most of the albumin was removed from the rabbit IgG polyclonal antibodies. Then, the ammonium sulfate was removed by dialyzing against 15 mM PBS for 3 days at 4 °C. The immunoglobulins were obtained from the supernatant and concentrated using polyethylene glycol [50]. Secondly, protein A–sepharose gel was used to purify rabbit IgG polyclonal antibodies, and a 0.1 M glycine buffer was used as the eluting buffer as described by the method of Abd El Hafez et al. [50]. The protein content of the IgG polyclonal antibodies was estimated following the Lowry et al. [47] method.

### 2.13. Assessment of Rabbit IgG Polyclonal Antibodies Reactivity Against GlAg Using Indirect ELISA

Indirect ELISA was applied to evaluate the reactivity of the purified IgG polyclonal antibodies according to Engvall and Perlmann [49]. The ELISA plate was coated with 4 μg/mL of specific GlAg overnight, washed 3 times, and blocked with 1% bovine serum albumin (BSA; PAN Biotech, USA). After that, two-fold serial dilutions of the IgG polyclonal antibodies were dispensed into wells. Anti-rabbit IgG horseradish peroxidase (HRP) conjugate (Sigma Chemical Co., St. Louis, MO, USA) was used. Ortho-phenylenediamine-dihydrochloride (OPD; Sigma-Aldrich) substrate was utilized to develop the color of the reaction. The absorbance of the optical density (OD) was measured at 450 nm using a microplate reader (Model EL×800UV, BioTek Instruments, Winooski, VT, USA).

### 2.14. Conjugation of Rabbit IgG Polyclonal Antibodies with HRP Enzyme

Labeling of the rabbit polyclonal antibodies (IgG) with HRP enzymes was performed mainly as described by Avrameas [51]. Briefly, 10 mg of HRP enzymes were mixed with 5 mg of IgG polyclonal antibodies in 1 mL total volume of 0.1 M phosphate buffer (pH = 6.8). The mixture was dialyzed over night at 4 °C against 0.1 M PBS (pH = 6.8). Using diluted glutaraldehyde, 50 μL was added to the dialyzed mixture with gentle stirring at room temperature for 3 h. Then, 2 M glycine solution was added to obtain a 0.1 M final concentration. The mixture was left at room temperature for 2 h and dialyzed overnight at 4 °C against 0.1 M PBS (pH = 6.8). Then, it was centrifuged for 30 min at 10,000× *g* at 4 °C. The supernatant was transferred into a sterile tube and one volume of glycerol was added. The conjugated antibody was stored at −20 °C in small aliquots until use.

### 2.15. Characterization of Rabbit IgG Polyclonal Antibodies and Crude GlAg

Electrophoretic analysis of the IgG polyclonal antibodies (anti-*Echinococcus* GlAb) and GlAg separately was performed in 10% polyacrylamide gels (SDS-PAGE) under reducing conditions according to Laemmli [52]. The relative molecular weights of the bands were calculated using Prestained molecular weight protein markers (GeneDirex BLUltra, USA) electrophoresed on the same gel. After separation, slab gel was stained with Coomassie Brilliant Blue dye, photographed, and analyzed using Molecular Imager with Image Lab Software (Gel Doc™ XR+, Bio-Rad, CA, USA).

### 2.16. Sandwich ELISA

An in-house Sandwich ELISA was performed to evaluate the sensitivity and specificity of the prepared IgG polyclonal antibodies, assess cross-reactivity with other infections, and detect the circulating *E. granulosus* antigens in the collected camel serum samples and compare them to the PM inspection. Briefly, to perform the Sandwich ELISA, 4 μg/mL of the optimal concentration of rabbit IgG polyclonal antibodies (anti-*Echinococcus* GlAb) was diluted 1:25 in coating buffer, and 100 μL/well was put into 96-well ELISA plates followed by incubation at 4 °C overnight. The excess antibody was removed by washing the plates three times in PBS-Tween 20 (0.1 MPBS, pH 7.4 containing 0.05% Tween 20). The wells were blocked with 200 μL/well of blocking buffer (1% BSA) and incubated for 2 h at 37 °C. They were then washed 3 times with PBS/T. Next, 100 μL/well of camel sera (diluted 1:100 with PBS) in duplicate from the infected camels confirmed as having CE, along with samples from healthy young camels used as negative controls, and other sera were added into the wells, and the plates were incubated for another 2 h at 37 °C. After a washing step, 100 μL/well of diluted 1:100 of horseradish peroxidase-conjugated IgG polyclonal antibodies (anti-*Echinococcus* GlAb) was added, and the plates were incubated for one and half hours at 37 °C. The plates were washed 5 times with washing buffer, the plates were then incubated with substrate buffer 100 μL/well (1 mg/mL ortho-phenylenediamine (Sigma-Aldrich), 0.025% H_2_O_2_ in 0.1 M citrate buffer, pH 5.0) for 20 min in the dark at room temperature, and then the reaction was terminated with a stopping solution of 50 μL/well (1 mM Sulfuric acid). The absorbance was measured using a microplate reader (EL×800UV, BioTek Instruments, USA). The cut-off was set as 3SD above the mean of the negative control samples [53].

Sensitivity, specificity, diagnostic efficacy, and accuracy percentages were estimated according to Parikh et al. [54] as follows: true-positive values (Tp): sera from camels naturally infected with *E. granulosus* as confirmed by parasitological examination; false-negative values (Fn): sera from camels infected with CE showing negative readings; false-positive values (Fp): sera from non-infected camels showing a positive result; and true-negative values (Tn): sera from healthy camels free of cysts as confirmed by veterinary inspection showing negative readings. Percentages were calculated using the following formulae:

Sensitivity% = Ntp/(NTp + NFn) × 100

Specificity% = Ntn/(NTn + NFp) × 100

Diagnostic Efficacy% = (NTn + NTp)/(NTp + NFp + NTn + NFn) × 100

Accuracy% = (NTn + NTp)/(NTp + NFp+ NTn + NFn) × 100

where N is the number of samples, Tp is the true positive, Fp is the False positive, Tn is the true negative, and Fn is the false negative.

In addition, the cross-reactivity with other parasitic infections other than CE, such as fasciolosis, ticks’ infestation, myiasis, coccidiosis, toxoplasmosis, and cryptosporidiosis was estimated in the presence of positive camels’ sera with cystic echinococcosis (with *E. granulosus*) and negative sera using Sandwich ELISA.

### 2.17. Statistical Analysis

Statistical analysis was performed using a SPSS version 19.0 for Windows (IBM Corp., Armonk, NY, USA). Data of cystic echinococcosis positive rates from PM, and Sandwich ELISA methods were analyzed using the Chi square test. *p < 0.05* was considered statistically significant. The diagnostic accuracy parameters of the test were evaluated by calculating the sensitivity, specificity, area under the curve (AUC), receiver operating characteristic (ROC) curve, and Chi square using SPSS software.

## 3. Results

### 3.1. Microscopical Examination and PM Diagnosis

**Figure 1:**
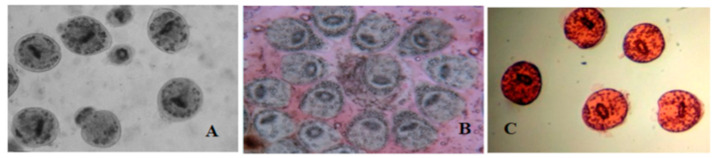
(**A**) Wet mount showing unstained hydatid protoscoleces of fertile hydatid cyst (×40). (**B**) Live colorless protoscoleces of fertile hydatid cysts after 5 min staining with 0.1% eosin (×40), live protoscoleces did not take the dye in and have normal color. (**C**) Dead protoscoleces of hydatid cysts after 5 min staining with 0.1% eosin (×40), dead protoscoleces take the dye in and appear red in color (×40).

Hydatid cysts were found in 273 infected camels out of the 587 examined camels (46.5%). Pulmonary echinococcosis was significantly more abundant in slaughtered camels (60%, 164/273) than hepatic CE (39.9%, 109/273), (*p* = 0.001, Chi Square = 11.081). Protoscoleces viability was determined using vital eosin 0.1% coloration (Figure 1). They were recorded in both liver visceral and parietal surfaces. The hydatid cyst fertility (reproducibility) was determined by the presence of free protoscoleces in the cystic fluid using a wet mount drop (Figure 1A). The fertility rates were higher in camel hepatic cysts (90.8%, 99/109) than in the pulmonary cysts (83.5%, 137/164). The most viable protoscoleces were recorded from fertile hepatic cysts (67.85 ± 12.78). Table 2 shows the fertility rates of the hydatid cysts and the viability of the protoscoleces from the fertile cysts in the liver and lungs of camels. The microscopical examination showed that the protoscoleces were seen as natural, bright, and colorless (Figure 1B) as the eosin dye (0.1%) could not penetrate the living protoscoleces, and the dead protoscoleces were shown as red in color (Figure 1C) as the dye could easily penetrate.

### 3.2. Standard PCR and Phylogenetic Analysis

**Figure 2:**
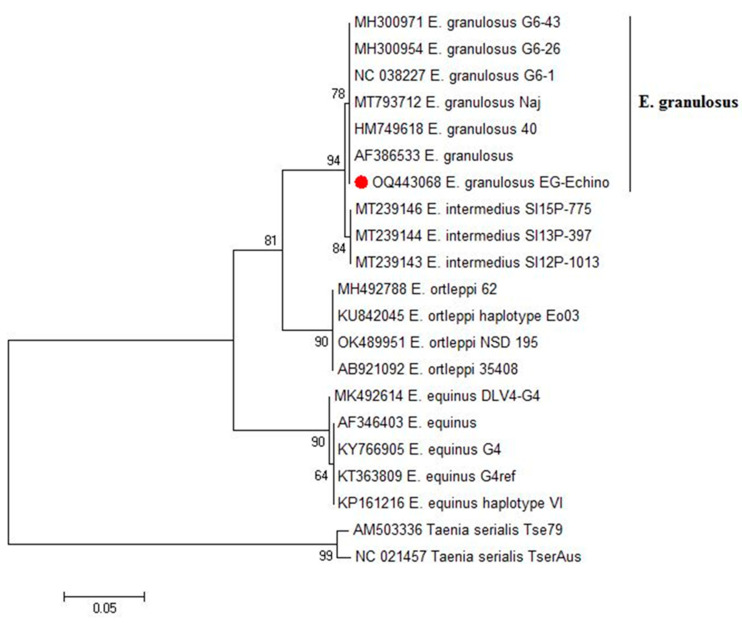
Phylogenetic analysis using the maximum likelihood method based on NAD1 gene for *Echinococcus* sp. The new obtained isolate sequence in this study is highlighted (red dot). There was a total of 435 positions in the final dataset. Our genotype clustered in a well-supported branch (bootstrap value 78) with other *E. granulosus* references. The scale bar represents a 5% nucleotide sequence divergence.

Ten DNA samples extracted from the protoscoleces collected from camels’ liver cysts provided the expected amplicon size (500 bp) for *Echinococcus* spp. In the gel electrophoresis after PCR using the NAD1 gene. The Blast analysis revealed that the presence of the genotype of *E. granulosus* in the investigated camels (GenBank: OQ443068.1). In the phylogenetic tree, our genotype clustered in a well-supported branch (bootstrap value 78) with other *E. granulosus* references (Figure 2).

### 3.3. Electrophoretic Profile of GlAg and Rabbit IgG Polyclonal Antibodies

**Figure 3:**
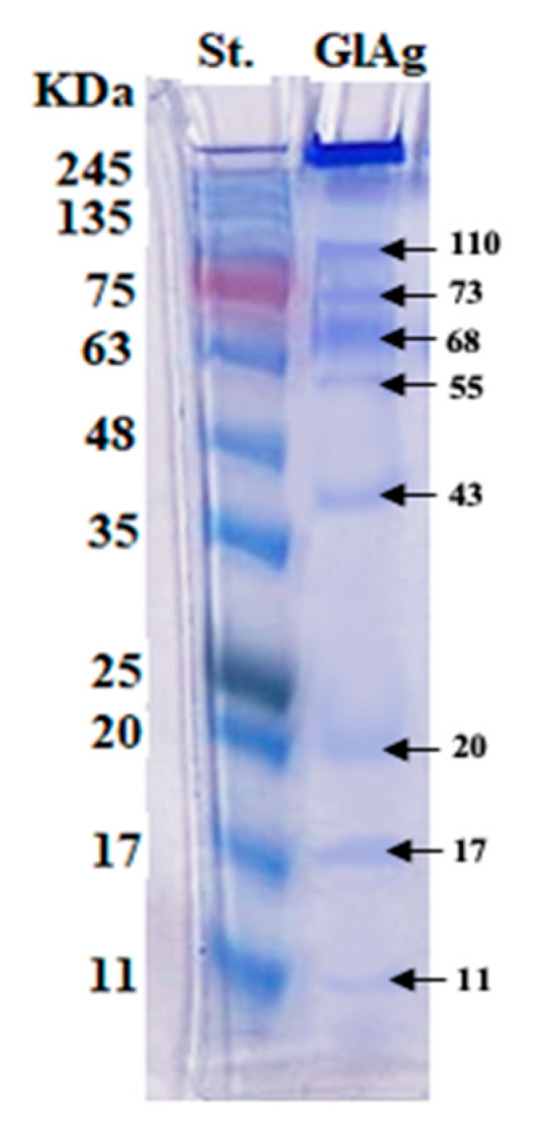
Lane GlAg; electrophoretic profile of the germinal layer antigen (GlAg), and Lane St.; prestained molecular weight protein markers ranging from 245 to 11 kDa.

**Figure 4:**
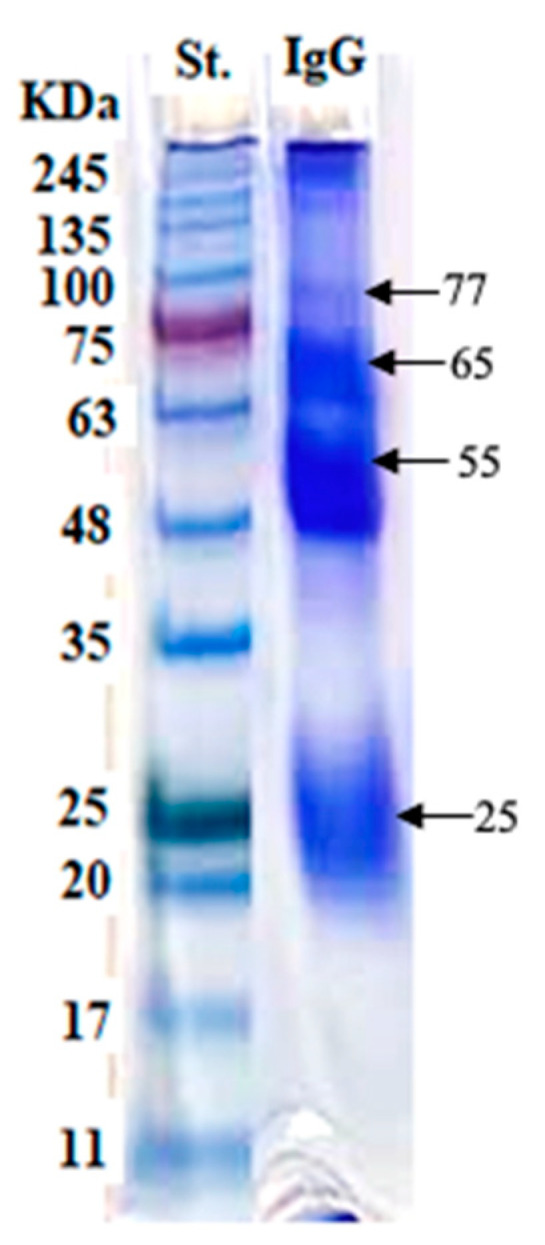
Lane IgG; electrophoretic profile of the purified rabbit IgG polyclonal antibodies (anti-*Echinococcus* GlAb), and Lane St.; prestained molecular weight protein markers ranging from 245 to 11 kDa.

Electrophoresis of the prepared crude GlAg showed multiple polypeptide bands at molecular weights of 110, 73, 68, 55, 43, 20, 17, and 11 kDa (Figure 3). Of the IgG polyclonal antibodies, three prominent bands were detected at 65, 55, 25 kDa, and a very faint band at 77 kDa, under reducing conditions in a 10% slab gel stained with Coomassie brilliant blue dye (Figure 4).

### 3.4. Diagnostic Potency of Rabbit IgG Polyclonal Antibodies against GlAg by Indirect ELISA

**Figure 5:**
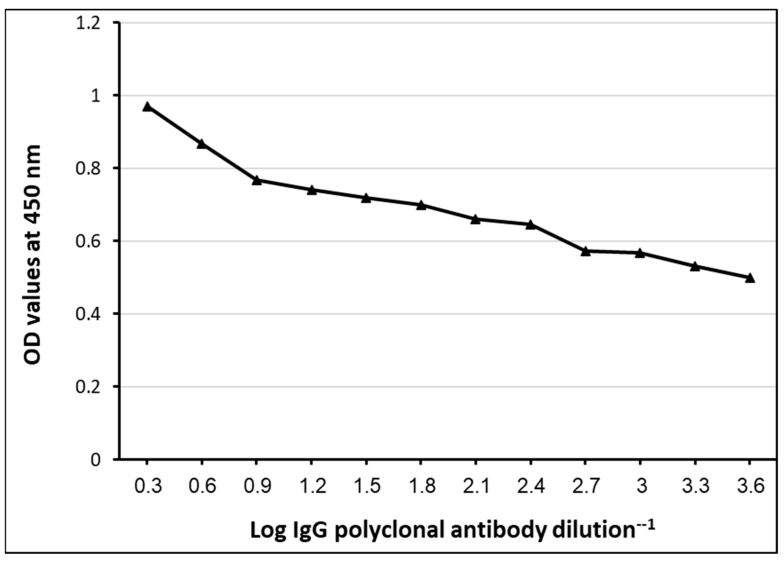
Potency of purified IgG polyclonal antibodies in reacting with GlAg by indirect ELISA at two-fold serially dilutions.

The results of the indirect ELISA showed that the purified IgG polyclonal antibodies gave a strong potent reactivity to GlAg with high sensitivity. While using two-fold serial dilutions (different dilutions) of purified IgG polyclonal antibodies, it succeeded in binding with GlAg, and the reaction still worked until a dilution of 1:4096 of purified IgG polyclonal antibodies (Figure 5).

### 3.5. Immunoreactivity and Cross-Reactivity of Rabbit IgG Polyclonal Antibodies for Detection of the Circulating E. granulosus Antigens in Camels’ Sera by Sandwich ELISA

The rabbit IgG polyclonal antibodies reacted strongly and detected 270/273 samples positive for CE. These three samples were among the light infection samples and the sensitivity of the assay was 98.9%. No cross-reactivity was recorded with the uninfected camel sera (negative sera for *E. granulosus*; healthy sera), all 57 healthy camel sera were below the cut off value (0.321) recording. However, the rabbit polyclonal antibodies IgG weakly reacted with three positive samples of camels’ fascioliasis (3/8), and 3/20 Myiasis (*C. titillator* larvae). These reactions indicated that there was a cross-reactivity between *F. gigantica, C. titillator,* and *E. granulosus*. Whereas, no cross-reactivity was found with samples of camels infested with ticks sp., or infected with *Eimeria* spp., *T. gondii,* and *Cryptosporidium* sp. Cross-reactivity compared to PM diagnosis is summarized in Table 3. The immunological diagnostic values of the Sandwich ELISA in the detection of *Echinococcus* GlAg in camels’ serum are shown in Table 4. Sensitivity was 98.9% (270/273), specificity was 94.9% (296/312), and the diagnostic efficacy was 96.8%. Regarding the ROC curve, the AUC was 0.99 (*p* < 0.001), revealing a high accuracy potential (0.9 > AUC > 1), Confidence Interval (0.991, 1.003), and Chi-Square = 587 (*p* < 0.001).

### 3.6. Detection of Circulating E. granulosus Antigen in Camel Sera for Diagnosis of CE by Sandwich ELISA

**Figure 6:**
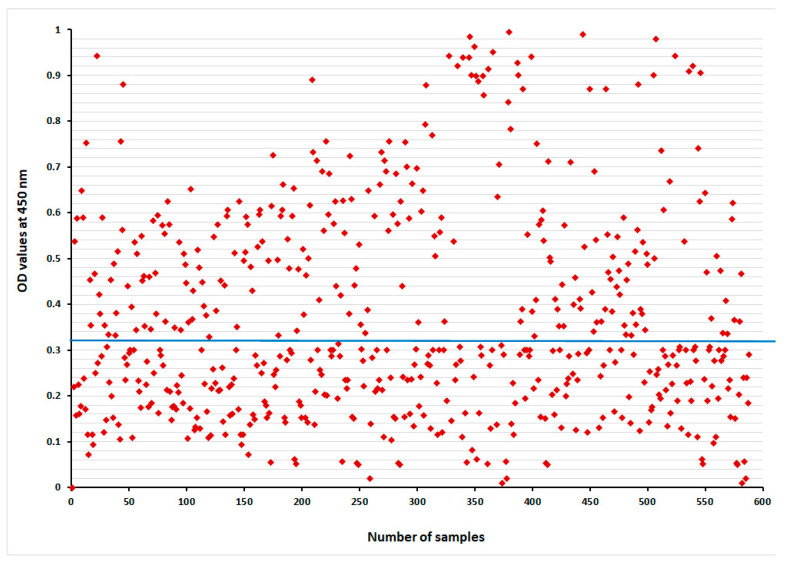
Diagnostic potency of rabbit IgG polyclonal antibodies in collected camel serum samples, blue line indicates cut off.

Sandwich ELISA was performed to test all the collected serum samples from the camels (*n* = 587) for *E. granulosus* antigen detection based on rabbit IgG polyclonal antibodies (anti-*Echinococcus* GLAb IgG). The serodiagnosis of camel sera using Sandwich ELISA assay resulted in a 48.7% (286/587) detection of the *E. granulosus* antigen in the collected camel sera at a cut off value = 0.321 OD (Figure 6). While the PM inspection resulted in a positive rate of CE in the same samples (46.5%, 273/587), (*p* = 0.5, Chi Square = 0.302).

## 4. Discussion

*E. granulosus* is an important zoonotic parasite, which has been recorded worldwide when carrying out veterinary inspections in slaughterhouses [8,55]. In this study, an in-house Sandwich ELISA procedure was developed using prepared rabbit IgG polyclonal antibodies from the hydatid germinal layers collected during a routine surveillance of CE in the main abattoirs of the Giza governorate. The results of our study showed that lungs and livers are mostly affected with hydatid cysts, as these organs are considered the first large capillary fields encountered by the blood-borne onchospheres [42,56]. This higher incidence of pulmonary echinococcosis than hepatic echinococcosis, reported in our study, might be attributed to the compact tissues of the liver, which may resist developing larger cysts [57]. On the other hand, lung parenchyma possesses a spongy consistency and a greater capillary bed that supports the wider distribution of onchospheres and larger embedded cysts [58]. We detected a higher rate of fertile and viable cysts in the hepatic cysts rather than in the pulmonary cysts, and this result agreed with Moudgil et al. [59] who recorded higher fertility rates in the hepatic cysts rather than in the pulmonary cysts of sheep. On the other hand, Haroun et al. [60] reported a lower percentage of fertile hydatid cysts (6.3%). These variations in fertility might be due to strain differences, pathogenesis, rate of development, infectivity, and resistance to drugs [61].

In the present study, the most viable protoscoleces were recorded from fertile hepatic cysts, which could be attributed to the route of parasite entry and that the hepatic portal distribution of onchospheres leading to liver infection [62]. Thus, we chose liver cysts for DNA extraction and molecular identification. PCR was performed to identify *Echinococcus* sp. and prepare the IgG polyclonal antibodies against antigens from a species common in camels. Isolate of *E. granulosus* recovered from hydatid cyst protoscoleces of naturally infected camels in our study were identical to those of the G6 *E. granulosus sensu lato* genotype strain detected in camels from Sudan (GenBank: MH300952.1), Mauritania (GenBank: MH300953.1 and MH300954.1), and Iran (GenBank: NC_038227.1) [63]. These results agreed with the previous reports, assuming that G6 is common in camels and is widespread in camel-raising countries of Africa, Asia, and the Middle East [64,65].

Indirect ELISA is considered a recommended assay for the detection of specific antibodies against CE in serum samples [29,66] with variable results of sensitivity and specificity [28,35,67]. This sensitivity and specificity rely on many factors, such as stability, quality, antigen source, and the used laboratory techniques [68,69]. Developing a reliable serological diagnosis in intermediate host animals is largely unsuccessful because of the coexistence of multiple infections of different taeniid species, cross-reactivity of antigens, and low-level responses of antibodies to infection [70]. So, antigen detection using the standard double antibody Sandwich ELISA in serum might be a promising alternative to measure the presence and/or concentration of circulating parasite antigens [30,39]. In our study, *E. granulosus* GlAg was utilized as a protein with significant immunological properties, having higher sensitivity and specificity in ELISA and a lower cross-reaction with antibodies of other parasites [35]. Our resulting molecular weight of the *E. granulosus* GlAg migrated into multiple bands, ranging from 110 kDa to 11 kDa on a reducing SDS-PAGE 10%. However, Kandil et al. [35] found that it migrated on a 12% SDS-PAGE at molecular weight ranges of 35 kDa to 150 kDa.

In our study, we performed two purification procedures for IgG GlAb, which were as follows: ammonium sulfate precipitation and protein A–sepharose gel. The resulting purified fraction of IgG GlAb was represented by four bands. Whereas El Deeb et al. [39] recorded that the purified rabbit anti-*E. granulosus* IgG protoscoleces (PAb) was resolved into two bands at 50 and 31 kDa assayed using 12.5% SDS-PAGE. This difference in molecular mass of purified rabbit anti-*E. granulosus* IgG might be attributed to the difference of the crude antigen used, the method of purification, and the percentage of the slab gel of the SDS-PAGE.

The purified IgG polyclonal antibodies gave a strong reactivity to GlAg as estimated by indirect ELISA. Sandwich ELISA, based on two capture (double) antibodies; the primary captures the purified IgG polyclonal antibodies coating the plate, and the secondary captures the GlAb IgG polyclonal conjugated with HRP. When using conduction Sandwich ELISA to detect for *E. granulosus* circulating antigen in camel serum samples, IgG polyclonal antibodies recorded high sensitivity and specificity. These results were higher than the protoscoleces antibodies (PAb) conjugated with gold nanoparticles for *E. granulosus* antigen detection using nano-gold dot-ELISA, which represented a sensitivity of 94.4% and a specificity of 90%, with an accuracy value of 92.9% of detection in human, camel, and sheep sera [71]. In addition, using Sandwich-ELISA to detect circulating *E. granulosus* antigens in human sera with PAb recorded a sensitivity of 90.5% and a specificity of 94.6% [39]. Moreover, Sadjjadi et al. [37] reported that the anti-hydatid cyst fluid IgG purified using protein A affinity chromatography gave a sensitivity of 25.7% and a specificity of 98%. These results proved that the standard double antibody Sandwich ELISA could be a promising method for the detection of *E. granulosus* circulating antigens.

The incidence of infection detected in our study using the Sandwich ELISA technique was higher compared to the PM examination data. This agreed with El-Baz [72] and Abo-Aziza et al. [73] who recorded higher results of CE serodiagnosis than in PM diagnosis. This might be attributed to small sized cysts, which might not be noticed by visual examination or the presence of cysts in other organs, which were not accurately investigated [72]. The high incidence obtained in our study might be due to the lack of suitable disposal of infected carcasses, presence of stray dogs, high number of disease reservoirs (camel, cattle, sheep, and goats), and lack of farmers’ awareness [56,74]. Serodiagnosis could be difficult due to the obstacles in the standardization of antigenic techniques and preparations [75,76].

## 5. Conclusions

In the present study, our designated Sandwich ELISA appeared to be sufficiently sensitive and specific for the detection of *E. granulosus* antigens in camel sera. Using IgG polyclonal antibodies could be added as an alternative for common immunodiagnostic tests available for the serodiagnosis of CE to help in the identification of CE infections during an animal’s life and facilitate treatment with drugs and/or control by slaughtering animals under special control measures to minimize the spread of infection to dogs. Also, conducting public campaigns is required to control CE by the elimination of stray dogs, proper disposal of infected organs, prohibiting the illegal slaughter of animals outside of abattoirs, fencing abattoirs, and increase people’s awareness on the disease epidemiology.

## Figures and Tables

**Table 1:** Blood samples collected from camels.

Blood Samples	Number
CE positive camel blood samples collected at PM inspection during several visits to abattoirs (gold standard positive control sera)	273
CE negative camel sera collected from healthy young camels, proposed to be free of cysts as confirmed by PCR, and other parasitic infections veterinary and PM inspection, and fecal examination (gold standard negative control sera)	57
Blood samples collected from slaughtered camels infected with *Fasciola gigantica* (Fascioliasis) in livers	8
Blood samples collected from camels infested with *Hyalomma dromedarii* ticks (Ticks are abundant on camels)	9
Samples collected from camels infested with *Cephalopina titillator* larvae at PM inspection (Myiasis, after examination of slaughtered camels’ skulls)	20
Samples from infected camels with Coccidiosis (Eimeriosis) caused by parasites of the genus *Eimeria* (as proved by fecal examination)	9
Infected camels’ sera with toxoplasmosis (positive anti-*Toxoplasma* antibodies detection in sera of camels by ELISA)	10
Blood samples from infected camels with *Cryptosporidium* sp. (as proved by fecal examination)	10
Blood samples from examined camels non infected with CE or other parasites	191
Total number of samples	587

CE: Cystic Echinococcosis; PM: Postmortem.

**Table 2:** Number of fertility hydatid cysts and viability of protoscoleces of fertile cysts recovered from different organs of slaughtered camels.

Animals Examined	Infected Organs Examined	Number of Hydatid Cysts/ Examined (%)	Number of Fertile Cysts (%)	Number of Sterile Cysts	Number of Calcified Cysts (%)	Viability of Protoscoleces in Fertile Cysts (Mean ± SD)
**Camels**	Lungs	164/273 (60%)	137 (83.5%)	18 (10.9%)	9 (5.5%)	55.23 ± 6.57
	Liver	109/273 (39.9%)	99 (90.8%)	6 (5.5%)	4 (3.7%)	67.85 ± 12.78

**Table 3:** Comparative serodiagnostic accuracy of Sandwich ELISA in the detection of circulating *Echinococcus* GlAg antigen in camel sera and PM diagnosis for detection of hydatid cysts in slaughtered camels.

Group (No. of Camel)	PM Diagnosis		Sandwich ELISA			
	Positive Number	Negative Number	Positive Serum Sample		Negative Serum Samples	
			No.	Optical Density Mean ± SD	No.	Optical Density Mean ± SD
Positive control sera infected with *E. granulosus* (*n* = 273)	273	0	270	1.055 ± 0.21	3	0.254 ± 0.03
Negative control for *E. granulosus*; healthy sera (*n* = 57)	0	57	0	0	57	0.138 ± 0.020
Camel Fascioliasis sera (*n* = 8)	0	8	3	0.345 ± 0.013	5	0.237 ± 0.015
Camel infested with *H. dromedarii* ticks (*n* = 9)	0	9	0	0	9	0.157 ± 0.017
Camel Myiasis (*n* = 20)	0	20	3	0.358 ± 0.021	17	0.218 ± 0.022
Camels with Coccidiosis (*n* = 9)	0	9	0	0	9	0.189 ± 0.031
Camel Toxoplasmosis (*n* = 10)	0	10	0	0	10	0.197 ± 0.013
Camel Cryptosporidiosis (*n* = 10)	0	10	0	0	8	0.246 ± 0.015
Camels non infected with CE or other parasites (191)	0	191	10	0.699 ± 0.21	181	0.248 ± 0.023

**Table 4:** The sensitivity, specificity, diagnostic efficacy, and accuracy of Sandwich ELISA in detection of *Echinococcus* GlAg in camels’ serum.

**Sandwich ELISA in Detection of *Echinococcus* GlAg**	**Sensitivity%**	**Specificity%**	**AUC/*p***	**Diagnostic** **Efficacy**	**Accuracy**
	98.9%	94.9%	0.99/<0.001	96.8%	96.8%

AUC: Area under curve.

## Data Availability

Data are available upon request.
